# Do gastrointestinal complaints increase the risk for subsequent medically certified long-term sickness absence? The HUSK study

**DOI:** 10.1186/1471-230X-11-88

**Published:** 2011-07-29

**Authors:** Simon Øverland, Marit Knapstad, Ingvard Wilhelmsen, Arnstein Mykletun, Nick Glozier

**Affiliations:** 1 Faculty of Psychology, University of Bergen, Bergen, Norway; 2 Institute of Medicine, University of Bergen, Bergen, Norway; 3 Division of Mental Health, National Institute of Public Health, Bergen, Norway; 4 Brain and Mind Research Institute, Sydney Medical School, University of Sydney, Sydney, Australia

**Keywords:** Sickness absence, gastrointestinal complaints, anxiety, depression

## Abstract

**Background:**

Gastrointestinal complaints are very common in the general population and very often co-occur with common mental disorders. We aimed to study the prospective impact of gastrointestinal complaints on long term sickness absence, and address the contribution from co-occurring common mental disorders and other somatic symptoms.

**Method:**

Health data on 13 880 40-45 year olds from the Hordaland Health Study (1997-99) were linked to national registries on sickness absence. As part of a wider health screening, gastrointestinal complaints were ascertained. Participant's anxiety and depression, and the presence of other somatic symptoms were evaluated. In Cox regression models, we predicted sickness absences over an average 5.4 years follow-up, with adjustment for confounders, anxiety and depression and other somatic symptoms.

**Results:**

After adjusting for gender, level of education and smoking, those reporting GI complaints had higher risk for later sickness absence (HR = 1.42, 95% CI 1.34-1.51). GI complaints were associated with both anxiety (OR = 3.66, 95% CI 3.31-4.04) and depression (OR = 3.28, 95% CI 2.89-3.72), and a high level of other somatic symptoms (OR = 8.50, 95% CI 7.69-9.40). The association of GI complaints was still independently associated with future sickness absence (HR = 1.17, 95% CI 1.10-1.16) adjusting for mental illness and other somatic symptoms.

**Discussion:**

Sickness absence is a complex behavioural outcome, but our results suggest GI complaints contribute by increasing the risk of long term sickness absence independently of comorbid mental illness and presence of other somatic symptoms. Occupational consequences of illness are important, and should also be addressed clinically with patients presenting with GI complaints.

## Background

Gastrointestinal (GI) complaints are very common in the general population, with, for example, more than half the sample of a large US-based study reporting such complaints in the previous three months [1]. In a Norwegian general population sample, 48% reported having had at least one GI complaint during the previous year [2]. Such complaints may be symptoms of distinct underlying organic pathology, but in many cases no clear explanation is found [3-7]. People with GI complaints, regardless of whether there is an underlying pathology, have a number of negative psychosocial sequelae, including a poorer quality of life [8,9] and increased health care utilisation [9-12]. These features are shared with somatic symptoms in general: They are very common [13,14], can often occur without a clear medical explanation [15], and are associated with distress and disability.

A major psychosocial consequence of illness across the OECD-member countries is the increasing proportions of the work force absent from work for longer periods of time. This leads to increased benefit expenditure, reduced productivity and tax income from the societal view and reduced income, loss of role and esteem for the individual [16]. Compared to the major societal and individual consequences of sickness absence [17] the lack of quality studies on the causes of sickness absence is striking [18], with few studies addressing this topic amongst people with GI complaints. Drossman and colleagues found that persons with GI complaints such as functional gastrointestinal disorder reported a higher number of days off work per year than the population free of such disorders [1]. A few other studies also provide indications for an association between GI complaints and self-reported sickness absence [19,20], but a recent Swedish study, found no such association between functional gastrointestinal disorders and sickness absences recorded in patient journals [21]. Only one study has evaluated this prospectively; a recent US study found that patients with functional dyspepsia had more short and long sickness absences than controls [22].

A number of cross sectional general population studies have reported strong associations between gastrointestinal complaints and both common mental disorders such as anxiety and depression [2,23-27] and general symptom reporting [28-30]. Mussel et al recently found that about one in five GI-patients in primary care also satisfy criteria for anxiety and depression [31], and a meta-analysis suggested that anxiety and depression are more common in people with both functional and verified gastrointestinal disorders than healthy controls [32]. As common mental disorders and somatic symptom reporting across organ systems are strong predictors for later awards of disability pension [33], any observed associations between GI complaints and sickness absence reported above may therefore be explained by these confounders. Further "lifestyle-factors" such as physical activity [34], smoking [35], alcohol use [36] and obesity [27,37] have all been linked to GI complaints and chronic physical illnesses. As these same factors also might relate to functional outcomes like sickness absence, they could explain parts of any association between GI complaints and sickness absence.

Given that the risk for permanent work force exit increases when sickness absence is long lasting [38] much policy is focussed upon those with long term sickness group. Efforts are being made to identify both high risk groups for this outcome and selected or indicated interventions to prevent it. Although there is a suggestion that people with GI complaints may be an important group to identify clinically the current literature on consequences of GI complaints seems to leave some important questions unanswered: Does reporting a high level of GI complaints at baseline predict long term sickness absences? If so, is it accounted for by one or more particular GI complaint? And, if such a predictive relationship is found, is it explained (in part or fully) by associated comorbidities or confounders measured at baseline?

## Methods

We conducted an historical cohort study employing data from a large population based health survey, linked to national registries of medically certified sickness absence benefits awarded up to 6.1 years after the baseline health survey.

### Population and data material

The Hordaland Health Study 1997-1999 (HUSK) was a joint epidemiological research project carried out by the National (Norwegian) Health Screening Service in collaboration with the University of Bergen. The base population included 29 400 individuals in Hordaland County in western Norway born 1953-57, aged 40-47 at the time of the data collection. Data were collected using two sets of questionnaires and clinical examinations. A total of 18 581 (8 598 men and 9 983 women) both answered the first questionnaire and came to the clinical examinations, yielding an initial participation rate of 63% (57% for men and 70% for women).

We excluded another 2 646 cases who did not return or complete needed items on the second questionnaire (administered at the clinical examination to be filled out and returned later), and another 1 875 who did not report being in paid work at baseline. A further 164 were excluded as their first short sickness absence period after health survey participation led directly to award of a permanent disability pension e.g. in the case of certain terminal or catastrophic illness. This left a final sample of 13 880 (approximately 47% of the base population).

### Exposure: Gastrointestinal complaints

In the first questionnaire participants were asked if they experienced each of six common gastrointestinal (GI) complaints ("stomach pain", "nausea", "feeling bloated", "coated tongue", "vomiting or regurgitation" and "frequent loose bowel movements", extracted from the ICD-10 research criteria for F45-Somatoform disorders [39]), "almost never", "rarely", "sometimes", "often", or "almost always", scored 0-4. For the main analysis, we were interested in identifying people who reported a high level of gastrointestinal complaints; We therefore summed each participant's total score across the 6 GI items, and as the distribution was highly skewed, we constructed a dichotomy with the 80^th ^percentile as cut off *(high level of GI complaints)*. To examine if the risk of sickness absence was confined to specific complaints, we constructed another set of variables where a response to each of the six items of "often" or "almost always" was dichotomously coded as 1, and less often as 0.

### Anxiety and depression

*Anxiety *and *depression *were assessed with the Hospital Anxiety and Depression Scale (HADS), which contain seven items each on cognitive symptoms of anxiety disorder and depression (HADS) [40]. In a recent literature review, HADS showed good case-finding properties for anxiety and depression in primary care patient populations [41]. A cut-off score of ≥8 on each subscale was found to give the optimal balance between sensitivity and specificity (both about 0.8) for depression and anxiety according to DSM-III and IV, or ICD-8 and -9 [41], and was therefore was therefore used as cut-off.

### Other somatic symptoms

The participants were also asked if they experienced each of the following 11 symptoms "almost never", "rarely", "sometimes", "often" or "almost always" (0-4): chest pain, breathlessness, dysuria, unpleasant sensations in or around the genitals, complaints of blotchiness or discolouration of the skin, unpleasant numbness or tingling sensations, joint or muscle pain (all derived from the ICD-10 research criteria for F45 - Somatoform disorders [39]), sore or running eyes or nose, headache, dizziness, fatigue. In line with identifying those with high GI complaints levels we constructed another variable identifying a general high level of somatic symptoms by summing these scores and dichotomised at the 80^th ^percentile.

### Physical conditions

*Physical conditions *were assessed through self-report in the form: "do you have or have you had any of the following", followed by a list of ten conditions: coronary infarction, angina, stroke, asthma, diabetes, multiple sclerosis, hay-fever, chronic bronchitis, osteoporosis or fibromyalgia. From previous studies it was clear that the prevalence of these conditions in this middle aged working population was low. We therefore dichotomised this into those with no conditions "0", and those with one or more conditions "1". Weight and height was measured by research nurses, and BMI categorised as normal (BMI < 24.9), overweight (BMI 25-29.9) and obese (BMI 30+).

### Demographics and health behaviours

The highest *education *level reported was recoded into four categories: "elementary schooling", "upper secondary school", "1-3 years of higher education" and "higher education exceeding four years". Information on *age *and *gender *was provided by the national population registry prior to invitation and inclusion in the health survey. Alcohol usage, assessed through self reported consumption of beer, wine and spirits over the past two-week period was categorised as abstinence, or low, medium and high consumption defined according to gender specific tertiles. *Physical activity *was measured through two variables on intense and light physical activity. These were combined into one variable reflecting "no", "moderate" and "high levels of physical activity". *Smoking *status was defined as daily smoker vs. other.

### Outcome: Sickness absence

Information on sickness absence awarded until end of 2003 was collected from the Norwegian National Insurance Administration, and merged with the HUSK data by Statistics Norway using national personal identification numbers. In Norway, the employers cover the first 16 days of a sickness absence (first 14 days until April 1998). After this, the National Insurance Scheme covers absences up to a total of 52 weeks. As a consequence, the official registries (which are used in the present study) do not include information on absences shorter than 16 (or previously 14) days. Further in the Norwegian system, a 56-day consecutive sickness absence prompts a thorough medical report including an activity plan for the patient's return to work. After 12 weeks, the national insurance scheme requires an extended plan and meetings towards the same purpose, which falls close to the previously used definition of long-term sickness absence of 90 consecutive days [42]. We therefore registered the first incident sickness absence from 17 days after the health survey participation, and used the start-stop dates to constructed the following mutually exclusive variables: i) The first LTSA (Long Term Sickness Absence) lasting from 17-55 consecutive days during follow-up, ii) The first LTSA lasting from 56-89 consecutive days during follow-up, and iii) The first LTSA lasting for more than 90 consecutive days during follow-up. As contrast for all these variables were those with no LTSA during follow-up.

### Statistical analysis

Descriptive statistics were reported as means and frequencies. We then examined if there were significant differences in the distribution of the potential confounding variables between participants with and without GI complaints, and those with or without sickness absence during the follow up period using independent sample t-tests for continuous variables and chi-square statistics for categorical variables. Due to the large sample size, differences may be significant but yet without practical importance. For all significant associations we calculated the effect size (Cohen's w [43]) and included variables in the multivariate models only if they were significantly associated to both exposure and outcome, and had at least a small effect size (w≥0.10) with either exposure or outcome. Logistic regression models (presented as odds ratios with 95% confidence intervals) were used to investigate the strength of associations between anxiety/depression/somatic symptoms and both the individual gastrointestinal symptoms, as well as high GI complaints. As we had exact information on the time between the baseline measurements and outcome data, we used Cox regression to estimate hazard ratios (with 95% confidence intervals) for later sickness absence from GI complaints, adjusted for confounding. We predicted risk for the first occurrence of long term sickness absence after the baseline health survey, while also taking into account length of this first period. In a hierarchical fashion these models were then adjusted for anxiety or depression, or experiencing a high load of other somatic symptoms. Finally, we examined if there were any additive interaction between GI complaints and anxiety, depression or gender towards risk of LTSA. All analyses, including identifying regression coefficients for the interaction analyses, was done in STATA 11, while the presence of additive interaction was examined using the algorithm suggested by Andersson et.al [44], where a synergy index (SI) deviating from "1" indicates presence of an additive interaction [45].

### Ethics

The study protocol was approved by the Regional Committee for Medical Research Ethics, Western Norway and by the Norwegian Data Inspectorate.

## Results

Higher levels of gastrointestinal complaints were observed amongst females, those with lower levels of education, the health risk behaviours of smoking, high BMI, and low levels of physical activity, and amongst those reporting physical illness, high physical symptom load and case levels of anxiety and depression. Only the associations with the last three variables were of sufficient effect for inclusion in the further analyses (table 1). The same variables had similar associations with the taking of at least one episode of sickness absence greater than 16 days during follow up (LTSA), with female gender, lower education and smoking being associated at the level set for inclusion in further analyses, in addition to high physical symptom load, anxiety and depression (table 2). Age and the level of alcohol use were not associated with either GI symptoms or LTSA.

**Table 1:** Total sample characteristics and associations with levels of GI complaints at baseline*

	Full sample		GI complaints <80th percentile		GI complaints >80th percentile			
	n	%	n	%	n	%	difference	Cohen's w**
Total sample	13880	100	11245	81.0	2635	19.0		
Age (mean/SD)***	43.2	1.5	43.2	1.54	43.2	1.56	t(-1.4), df = 13878, p = 0.17	-
Gender							χ^2 ^= 74.9, df = 1, p < 0.001	0.07
Males	6694	48.2	5623	50.0	1071	40.7		
Females	7186	51.8	5622	50.0	1564	59.4		
Highest education level							χ^2 ^= 75.0, df = 2, p < 0.001	0.07
Elementary school	2226	16.2	1688	15.1	538	20.6		
Upper secondary school	6367	46.2	5111	45.8	1256	48.0		
Higher education	5189	37.7	4368	39.1	821	31.4		
Physical illness	1010	7.3	741	6.6	269	10.2	χ^2 ^= 41.7, df = 1, p < 0.001	0.05
BMI							χ^2 ^= 22.7, df = 2, p < 0.001	0.04
Normal (BMI < 25)	7083	51.1	5776	51.4	1307	49.7		
Overweight (BMI 25-30)	5296	38.2	4322	38.5	974	37.0		
Obese (BMI >30)	1491	10.8	1140	10.1	351	13.3		
Smoking	4705	33.9	3658	32.5	1047	39.7	χ^2 ^= 49.4, df = 1, p < 0.001	0.06
Alcohol use							χ^2 ^= 2.2, df = 3, p = 0.54	-
Abstainer	1027	7.6	828	7.5	199	7.7		
Low consumption	4431	32.7	3577	32.5	854	33.1		
Average consumption	4287	31.6	3504	31.9	783	30.4		
High consumption	3823	28.2	3082	28.0	741	28.8		
Physical activity							χ^2 ^= 30.8, df = 2, p < 0.001	0.04
No activity	2103	15.3	1629	14.6	474	18.2		
Moderate	5622	40.8	4526	40.6	1096	42.0		
High	6041	43.9	5002	44.8	1039	39.8		
Anxiety	2281	16.4	1376	12.2	905	34.4	χ^2 ^= 759.8, df = 1, p < 0.001	0.23
Depression	1186	8.5	718	6.4	468	17.8	χ^2 ^= 353.5, df = 1, p < 0.001	0.16
Somatic symptoms	2375	17.1	1090	9.7	1285	48.8	χ^2 ^= 2300.0, df = 1, p < 0.001	0.41

* For dichotomous variables we present numbers and rates for positive cases only

** Effect sizes calculated for significant univariate associations only

*** Continuous variable: presented with mean and standard deviations (SD) and test for differences with independent sample t-test.

**Table 2:** Association of baseline characteristics with Long Term Sickness Absence (LTSA) over up to 6 years of follow up*

	No LTSA during follow up		One or more period of LTSA during follow up			
	n	%	n	%	difference	Cohen's w**
Total sample	7422	53.5	6458	46.5		
Age (mean/SD)***	43.1	1.54	43.2	1.55	t(-1.8), df = 13878, p = 0.08	-
Gender					χ^2 ^= 304.7, df = 1, p < 0.001	0.15
Males	4444	54.4	2250	39.4		
Females	3722	45.6	3464	60.6		
Highest education level					χ^2 ^= 265.2, df = 2, p < 0.001	0.14
Elementary school	1058	13.0	1168	20.6		
Upper secondary school	3592	44.3	2775	49.0		
Higher education	3465	42.7	1724	30.4		
Physical illness	500	6.1	510	9.0	χ^2 ^= 39.3, df = 1, p < 0.001	0.05
BMI					χ^2 ^= 26.4, df = 2, p < 0.001	0.04
Normal (BMI < 25)	4194	51.4	2889	50.6		
Overweight (BMI 25-30)	3182	39.0	2114	37.0		
Obese (BMI >30)	787	9.6	704	12.3		
Smoking	2460	30.1	2245	39.3	χ^2 ^= 126.0, df = 1, p < 0.001	0.10
Alcohol use					χ^2 ^= 17.3, df = 3, p = 0.54	-
Abstainer	578	7.2	449	8.1		
Low consumption	2535	31.6	1896	34.1		
Average consumption	2618	32.7	1669	30.0		
High consumption	2281	27.5	1542	27.8		
Physical activity					χ^2 ^= 32.2, df = 2, p < 0.001	0.05
No activity	1155	14.2	948	16.8		
Moderate	3255	40.1	2367	41.9		
High	3712	45.7	2329	41.3		
Anxiety	1129	13.8	1152	20.2	χ^2 ^= 98.3, df = 1, p < 0.001	0.08
Depression	591	7.2	595	10.4	χ^2 ^= 43.4, df = 1, p < 0.001	0.06
Somatic symptoms	1019	12.5	1356	23.7	χ^2 ^= 300.1, df = 1, p < 0.001	0.15

* For dichotomous variables we present numbers and rates for the positive cases only

** Effect sizes calculated for significant univariate associations only

*** Continuous variable: presented with mean and standard deviations (SD) and test for differences with independent sample t-test.

Each of the individual GI symptoms was statistically significantly associated with anxiety and depression, with odds ratios ranging from 2.29 to 4.63 (table 3). The strongest association was found between anxiety and nausea (OR 4.63, 95% CI 3.38-6.34). While some of the specific GI complaints were more strongly associated with anxiety and depression than others, their respective associations with anxiety and depression were similar with overlapping confidence intervals. For each of the specific GI-symptoms, the association with a general high level of somatic symptom reporting was stronger compared to that for anxiety and depression.

**Table 3:** Gender, education and smoking adjusted associations between the GI complaints individually and combined, and anxiety, depression and other somatic symptoms

	Nausea		Stomach pain		Feeling bloated		Coated tongue		Vomiting or regurgitation		Frequent loose bowel movements		GI complaints (>80th percentile)	
	OR	95% CI	OR	95% CI	OR	95% CI	OR	95% CI	OR	95% CI	OR	95% CI	OR	95% CI
Anxiety	4.63	3.38-6.34	3.23	2.75-3.80	2.56	2.27-2.88	2.42	2.09-2.81	3.02	2.30-3.97	2.63	2.26-3.07	3.66	3.31-4.04
Depression	4.43	3.10-6.32	3.19	2.63-3.87	2.56	2.20-2.97	2.29	1.89-2.76	2.79	2.02-3.84	2.55	2.12-3.06	3.28	2.89-3.72
Somatic symptoms	7.20	5.19-9.99	4.75	4.04-5.56	4.14	3.70-4.64	4.60	4.00-5.30	4.93	3.78-6.44	3.80	3.28-4.42	8.50	7.69-9.40

The presence of high levels of GI complaints was associated with future LTSA (table 4). The overall risk for any LTSA over follow up, adjusted for gender, education and smoking was 1.42 (95% CI 1.34-1.51), and 1.17 (95% CI 1.10-1.16) in the fully adjusted model. When comparing subgroups of LTSA defined by duration, the hazard ratios were higher with longer durations of the first period of LTSA after the health screening, although with overlapping confidence intervals: For LTSA lasting 17-55 consecutive days, the gender, education and smoking adjusted risk from GI complaints was 1.47 (95% CI 1.34-1.60), while the corresponding risk if the first period lasted 90 days or more was 1.70 (95% CI 1.51-1.93). Individual further adjustment for depression explained between 7 and 8% of the risk, while adjustment for anxiety explained between 10 and 17% of the risk. Adjustment for potential confounding of general somatic symptom reporting on top of gender, education and smoking explained a larger proportion of the risk: between 32 and 48%. In the final model including all covariates simultaneously, the risk was substantially attenuated but still statistically significant at 1.19 (95% CI 1.08-1.32) for LTSA's between 17-55 days and 1.33 (95% CI 1.16-1.53) for 90 or more days (table 4).

**Table 4:** Cox proportional hazard models assessing the association of high levels of GI symptom reporting with subsequent long term sickness absence (LTSA) of various durations, with adjustments for individual potential confounders and finally adjusting for all potential confounders

	LTSA >16-55 consecutive days (predicting 2763 LTSA's)		LTSA >55-89 consecutive days (predicting 1258 LTSA's)		LTSA >89 consecutive days (predicting 1340 LTSA's)	
Adjustments	HR	95%CI	HR	95%CI	HR	95%CI
Crude	1.59	1.46-1.74	1.72	1.51-1.96	1.91	1.69-2.16
Model 1: Gender, education and smoking	1.47	1.34-1.60	1.55	1.36-1.77	1.70	1.51-1.93
Model 1 + depression	1.43	1.31-1.57	1.50	1.31-1.71	1.63	1.44-1.85
Model 1 + anxiety	1.40	1.27-1.53	1.48	1.29-1.70	1.61	1.42-1.83
Model 1 + somatic symptoms	1.22	1.10-1.34	1.35	1.17-1.56	1.38	1.20-1.58
Full adjustment*	1.19	1.08-1.32	1.31	1.13-1.52	1.33	1.16-1.53

* Adjusted for gender, education, smoking, depression, anxiety and somatic symptoms.

With regard to specific GI complaints, stomach pain had the strongest gender, education and smoking adjusted association with LTSA (HR = 1.69, 95% CI 1.53-1.87). The variable "coated tongue" had the weakest initial risk with HR = 1.35 (95% CI 1.23-1.48). After adjustment for other symptoms, nausea and coated tongue no longer incurred any independent risk of LTSA (table 5).

**Table 5:** Cox proportional hazard models assessing the association of high levels of GI symptom reporting with any subsequent long term sickness absence (LTSA) with adjustments for individual potential confounders and finally adjusting for all potential confounders

	Nausea		Stomach pain		Feeling bloated		Coated tongue		Vomiting or regurgitation		Frequent loose bowel movements	
	HR	95%CI	HR	95%CI	HR	95%CI	HR	95%CI	HR	95% CI	HR	95%CI
Crude	1.65	1.34-2.02	1.69	1.53-1.87	1.51	1.41-1.63	1.35	1.23-1.48	1.60	1.35-1.90	1.38	1.25-1.52
Adjusted for gender, education and smoking	1.42	1.16-1.75	1.57	1.42-1.74	1.38	1.28-1.49	1.27	1.16-1.40	1.59	1.34-1.89	1.42	1.29-1.57
Anxiety*	1.28	1.05-1.58	1.46	1.32-1.62	1.32	1.22-1.42	1.21	1.10-1.33	1.47	1.23-1.75	1.34	1.22-1.48
Depression*	1.33	1.08-1.63	1.51	1.36-1.67	1.34	1.25-1.44	1.24	1.13-1.37	1.51	1.27-1.80	1.37	1.24-1.51
Somatic symptoms*	1.14	0.93-1.40	1.35	1.21-1.49	1.21	1.12-1.31	1.09	0.98-1.20	1.34	1.13-1.60	1.25	1.13-1.38

* Adjusted for the health variable on top of gender, education and smoking.

Finally, we did not identify any significant additive interactions between GI complaints and anxiety (SI = 1.10, 95% CI 0.84-1.42), depression (SI = 1.09, 95% CI 0.78-1.53), or gender (SI = 1.06, 95% CI 0.89-1.27), in predicting LTSA.

## Discussion

### Main findings

In this large population based cohort study, people who reported high levels of GI complaints were at increased risk of long-term sickness absence (>16 days in the Norwegian system) over up to 6 years later. As in previous studies, there was a strong association between anxiety and depression and a high level of GI complaints, particularly nausea. Despite this, anxiety and depression explained relatively little of the increased risk for sickness absence arising from GI complaints. An overall high level of other somatic symptoms explained comparatively more of the risk. These results were similar for any one of the specific GI complaints.

### Strength and weaknesses

The main strengths of this study lie in its prospective nature, size, ability to adjust for multiple confounders, and that measurement at baseline could not be biased with regards to the aim of this study. Furthermore our combined use of health study data and objective information on sickness absences from public registries reduces common method problems. The payment of benefits requires correct registration and a personal identification number, and for this reason the outcome data are considered highly accurate. Only people leaving Norway or dying would be excluded from follow up.

However the final participation rate with full data was only 47%. Results from a recent study suggest that non-participation in Norwegian population based health studies probably lead to underestimated prevalence estimates, but that studies focusing on associations between variables suffers less from health selection in non-participation [46]. In addition non-participation is higher amongst sicker people, those with mental disorder and those with higher rates of LTSA. Thus our observations would likely be an underestimate of any true association.

The measurement of GI symptoms was taken from a somatisation assessment. This did not include any measure of duration of symptoms, unlike the Rome III criteria for functional gastrointestinal disorders, which separate the chronic and the fluctuating conditions. Our lack of a duration criteria is a weakness as we are modelling the associations with long-term outcomes that, if indeed caused by the GI complaints, should be limited to the chronic or recurrent cases. Again this weakness should lead us to present underestimations of the true association.

In previous papers using the same variable on somatic symptoms, including the GI symptoms, we have employed missing substitutions using individual mean substitution assuming "missing at random". We did not go to any such steps for the GI items for this paper, as this would inflate the correlations between the GI complaints and other symptoms.

The health study did not include any of the clinical information required for excluding organic aetiology for the GI complaints presented by the participants in the present study. Other physical conditions, which are adjusted for, and medications taken should in theory be only partly related to GI complaints, as supported in our initial univariate analysis on this association. This is a clear limitation as the GI complaints could be symptoms of underlying pathology. Below, we discuss the relevance of this for clinical management.

### Interpretation

The key finding in the present study was the increased risk of LTSA during follow-up among those with a higher level of GI complaints. The most parsimonious interpretation of this is that these GI complaints are symptoms or a marker for a range of underlying gastrointestinal pathologies. In this study, we had no capacity to examine possible organic causes for these GI complaints. At the same time, we know that much of the time such complaints are not explained by positive findings [5,6,15,47,48] and in a non-clinical sample of people in their forties, functional complaints should be more common than organic failure or pathology. Supporting this are the observations that although nearly 14 000 individuals provided answers to the items of interest for the present study, this is still only about 47% of the approximately 29 000 eligible 40-47 year olds in the county at the time of the health study. Those who did not participate had poorer average health [49,50] and more often received benefits [51].

Another explanation could be that that these symptoms are expressions of the most common causes of long term sickness absence: depression and anxiety. Certainly we confirmed a strong association between GI complaints and anxiety/depression. However adjusting for these potential confounders did little to explain the observed association. The overall measure of physical symptom reporting was both highly associated with GI complaints and LTSA and appeared to be a strong confounder. In this study relatively few participants (7.3%) had a chronic physical illness, and only a few of these illnesses in the list above would have resulted in GI complaints. In some cases, these other somatic symptoms may well be followed by gastro-related organic failure. Adjusting for the other symptoms could reflect overadjustment, leading us to underestimate the impact of the GI complaints on sickness absence. This latter is in line with Agreus' study where those with GI complaints had more sickness absence than the general population, but their sickness absences were most often warranted from non GI-related medical causes [20]. While this certainly is possible within such a large sample, several factors suggest this should not be a major factor. The GI complaints were also abstracted from a list of symptoms that in sum makes up the requirements for somatisation disorder. Splitting these symptoms into organ specificity, and then reintroducing the remaining symptoms as adjustments, may by default introduce over-adjustment of associations. Taken together, these findings could indicate that although GI complaints are related to anxiety and depression, the functional outcomes from GI complaints in terms of sickness absence are possibly due in large part to GI complaints being part of a person's tendency to experience and/or report symptoms across the various organ systems. This hypothesis is supported by a previous paper from this cohort showing that high levels of health anxiety was a strong predictor of leaving the workforce entirely and moving onto a disability pension [52].

The discrepancy between "explained" and "unexplained" or "functional" gastrointestinal conditions is blurred and changing with new developments in e.g. understanding of pain and neuropathology. For some psychosocial outcomes the distinction may be less relevant: A recent study by Kisely and colleagues [53] found that the difference in functional outcomes between explained and unexplained symptoms were rather small. In addition, there seems to be a relatively low correspondence between organic findings and the degree of suffering from the symptoms [54,55]. Finally, welfare schemes influence access to sickness absences, and the Norwegian system is known as relatively generous. Still, studies from US populations also suggest GI complaints are associated with occupational consequences [1,22], and the associations between GI complaints, other symptoms and mental illnesses, is consistent in the international literature. Our main finding of an independent effect of GI complaints should therefore also be informative beyond a Norwegian context.

## Conclusions

A high level of gastrointestinal complaints predicts objectively ascertained long term sickness absences. This was consistent across varying definitions of long term sickness absence. Our results confirmed the close relationships between GI complaints, depression and anxiety, but at the same time this did not seem to explain the work related functional outcomes of GI complaints. The presence of other somatic symptoms seems more important in understanding functional outcomes of GI complaints, lending support to theories of commonalities across symptom representations. Future work using more advanced latent class and path analytic techniques will help our understanding of how these symptom patterns combine and contribute to complex behaviours such as sickness absence.

For clinicians our results would suggest that management of the investigation and treatment of any underlying pathology in those with GI complaints should continue to be augmented by helping the individual manage their behaviour and disability. This is not just a focus on identifying and treating comorbid psychological illness, which is important, but would involve a nuanced understanding of individual's beliefs about their symptoms, the causes and implications. Identifying maladaptive behavioural responses to GI symptoms may help people improve their psychosocial outcomes. In this respect the work concerning illness perceptions, and demonstrations that tackling these can improve work related outcomes [56] may prove fruitful for clinicians and rehabilitation providers. Finally, our data do not pinpoint which aspects of GI complaints lead to long term absence from work. It could be the activity limitations associated with symptoms such as pain, or that, anecdotally, people often take time off work whilst they are being investigated. Beliefs that work somehow contributes to or perpetuates these symptoms, or may be a cause of a disease, can also contribute to people wanting sickness absence. It may even be that common factors such as early childhood experiences can explain the association [57,58]. Future clinical studies could benefit from including sickness absence as an outcome of interest, as it is an outcome of high societal and individual relevance, and help identify which aspects of gastrointestinal conditions lead to these poorer sequelae.

## Competing interests

The authors declare that they have no competing interests.

## Authors' contributions

SØ and MK planned the study, carried out analyses and drafted the manuscript. IW, AM and NG contributed to interpretation of results and revised the manuscript for important content. All authors read and approved the final manuscript.

## Pre-publication history

The pre-publication history for this paper can be accessed here:

http://www.biomedcentral.com/1471-230X/11/88/prepub
